# New Antifeedant Grayanane Diterpenoids from the Flowers of *Pieris formosa*

**DOI:** 10.3390/molecules22091431

**Published:** 2017-08-31

**Authors:** Chun-Huan Li, Shi-Hong Luo, Sheng-Hong Li, Jin-Ming Gao

**Affiliations:** 1 Shaanxi Key Labotory of Natural Products & Chemical Biology, College of Chemistry & Pharmacy, Northwest A & F University, Yangling 712100, China; chunhuanli@nwsuaf.edu.cn; 2 State Key Laboratory of Phytochemistry and Plant Resources in West China, Kunming Institute of Botany, Chinese Academy of Sciences, Lanhei Road 132, Kunming 650201, China; lsh246967@126.com

**Keywords:** *Pierisformosa*, Ericaceae, grayanane diterpenoids, antifeedant activity

## Abstract

Three new grayanane diterpenoids, pierisoids C‒E (**1**–**3**), as well as 10 known ones (**4**–**13**), were evaluated from the flowers of *Pieris formosa*, which is used as an insecticide in rural areas of China. Their structures were elucidated on the basis of extensive 1D and 2D NMR spectroscopic data analyses. Significant antifeedant activity of **1**, **3** and **10** against the beet armyworm (*Spodoptera exigua*) was found, indicating that these diterpenoids might also be involved in the plant defense against insect herbivores.

## 1. Introduction

**Figure 1:**
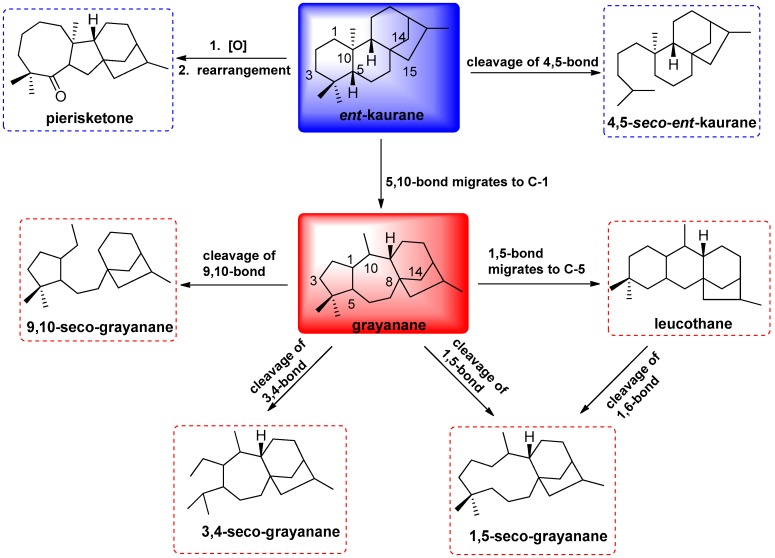
Biogenetic relationships of grayanane carbon skeletons from the genus *Pieris*.

Grayanoids represent a special type of diterpenoids, which have been limited to the plants of Ericaceae, such as the genera *Pieris*, *Rhododendron*, *Kalmia*, *Craibiodendron* and *Leucothoe* [1,2]. The grayanoid diterpenoids, as the characteristic secondary metabolites of the plants of Ericaceae, have attracted much attention from chemists and biologists not only for their intriguing structure but also for their diverse bioactivity, especially for their toxicity, as well as their analgesic, significant antifeedant, and insecticidal activity [3,4,5]. To date, 15 types of diterpenoid skeleton have been reported, including grayanane (A-nor-B-homo *ent*-kaurane) [5], 1,5-secograyanane [6], 3,4-secograyanane [7], 9,10-secograyanane [8], 1,10:2,3-disecograyanane [9], leucothane (A-homo-B-nor grayanane) [10], kalmane (B-homo-C-nor grayanane) [11], 1,5-secokalmane [12], micranthane (C-homo grayanane) [13], mollane (C-nor-D-homo grayanane) [14], rhodomollane (D-homo grayanane) [15], *ent*-kaurane, 4,5-*seco*-*ent*-kauran [16], pierisketane (A-homo-B-nor-*ent*-kaurane) [17], and rhodomollane [18]. All of these diterpenoid types are assumed to be derived biogenetically from the *ent*-kanrane skeleton. Notably, eight types of skeletons have been produced by the narrow genus *Pieris*, whose representative plant is *Pieris formosa* D. Don. (Figure 1).

**Figure 2:**
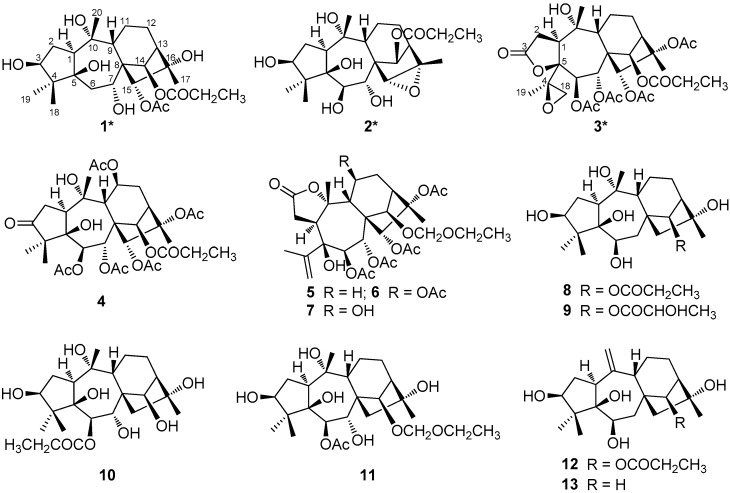
Structures of grayanane diterpenoids **1–13** isolated from *P. formosa*.

*P. formosa*, an evergreen shrub, is distributed mainly in the hilly regions and valleys of southern and southwestern China. The juice of both its fresh leaves and flowers are used as an insecticide and lotion for the treatment of ring worm and scabies, in folk medicine [19,20,21]. More than 60 grayanane diterpenoids with grayanane, 1,5-secograyanane, 3,4-*seco*-grayanane, 9,10-*seco*-grayanane, leucothane, 4,5-*seco*-*ent*-kauran, and pierisketane carbon skeletons have been isolated from the leaves, flowers, fruits, stems and roots of *P. formosa* [3,17,22,23,24,25]. In our previous work, two new highly esterified 3,4-*seco*-grayanane diterpenoids, pierisoids A and B, were reported from the flowers of *P. formosa* [20]. In our continuing endeavor to identify structurally unique and biologically diverse grayanoids from famous poisonous plants, three additional new grayanoids, pierisoids C‒E (**1**‒**3**) (Figure 2), together with ten known ones (**4**‒**13**) (Figure 2) were isolated from the flowers of *P. formosa*. Herein, the isolation and structural elucidation of **1**‒**3** and their antifeedant activity against the generalist plant-feeding cotton bollworm are described.

## 2. Results

### 2.1. Structural Elucidation of Compounds ***1**–**3***

Compound **1**, ${\left[\alpha \right]}_{D}^{24.8}$ = +14.3 (*c* = 0.2, MeOH), was isolated as colorless crystals. Its molecular formula was determined to be C_25_H_40_O_9_ according to its high-resolution (HR) ESI-MS (found: *m/z* 507.2567 [M + Na]^+^, calcd.: 507.2570) and ^1^H- and ^13^C-NMR spectra. IR absorptions at 3550, 3484, 1752 and 1729 cm^−1^ were indicative of hydroxyl and ester carbonyl functional groups. In the ^1^H-NMR spectrum (Table 1), four tertiary methyls at δ_H_ 0.91, 1.11, 1.27 and 1.41 (each 3H of singlet), one acetyl methyl at δ_H_ 2.10 (s, 3H), and one primary methyl at δ_H_ 1.11 (3H, t, *J* = 7.6 Hz) were clearly observed. Additionally, four singlets (δ_H_ 3.06, 3.39, 4.93 and 5.59), two triplets (δ_H_ 3.60 and 3.62, *J* = 5.0 Hz), and one doublet (δ_H_ 4.20, d, *J* = 5.4 Hz) were ascribable to either oxygenated methine or free hydroxyl groups. Other signals which were mostly overlapped centered between 1.58 and 2.85 ppm, resonating from either methine or methylene signals. The ^13^C-NMR spectrum revealed 25 carbon resonances, which were further classified by DEPT-90 and DEPT-135 spectra as six methyls, five methylenes, seven methines including four oxygenated ones (δ_C_ 72.4, 82.1, 82.5, and 85.0), seven quaternary carbons including three oxygenated ones (δ_C_ 78.4, 79.6, and 84.1), and two carbonyl carbons (δ_C_ 170.3 and 173.4). By analysis of the HSQC spectral data, all proton signals, except for the two singlets at δ_H_ 3.06 and 3.39 and the doublet at δ_H_ 4.20, could be assigned unambiguously to their respective carbons, suggesting that the signals at δ_H_ 3.06, 3.39 and 4.20 were assignable to free hydroxyl groups. Moreover, the existence of a propionyloxy-group was determined from analysis of the ^1^H-^1^H COSY and HMBC spectra. The above spectroscopic evidence suggested a highly oxygenated grayanane diterpenoid for **1**.

**Figure 3:**
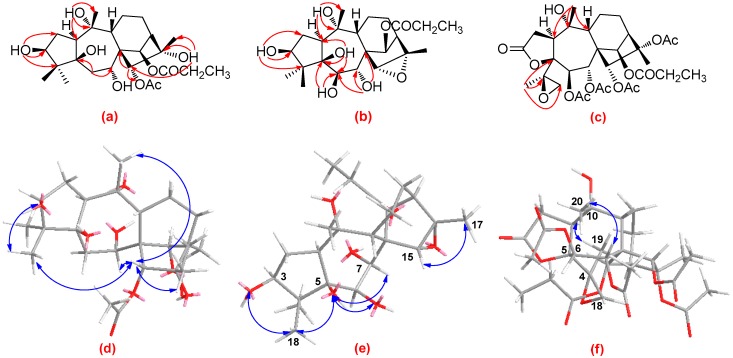
(**a**)–(**c**) are key HMBC correlations of pierisoids C–E, respectively; (**d**)–(**f**) are selected ROESY correlations of pierisoids C–E, respectively.

Detailed analysis of the 1D (^1^H and ^13^C) and 2D (^1^H-^1^H COSY, HSQC, and HMBC) NMR spectra (Figures S1–S6 in supplementary data) of **1** revealed that its structure closely resembled that of asebotoxin VIII [19,26], a known grayanane diterpenoid previously isolated from both *P. japonica* and *P. formosa*. The obvious difference between the two compounds was that the acetoxy group located at C-6 in asebotoxin VIII migrated to C-15 in **1**, as indicated by the HMBC correlations from H-15 (δ_H_ 4.93, s) to the acetoxy carbonyl group at δ_C_ 170.3 and from H_2_-6 (δ_H_ 2.05, m) to C-5 (δ_C_ 84.1) and C-7 (δ_C_ 72.4) (Figure 3a). In the ROESY spectrum, the correlations of H-15 with Me-18 as well as Me-20 suggested that H-15, Me-18, and Me-20 were all in the same β-orientation (Figure 3d). In addition, the correlations of 3-OH with Me-18, and of 15-H with 7-H and Me-17 suggested that 3-OH, H-7 and Me-17 were also in β-orientation (Figure 3d). Further analysis of the ROESY spectrum indicated the configurations of the remaining functional groups in **1** were the same as those in asebotoxin VIII, namely 3β, 5β, 7α, 10α, 16α-pentahydroxy, and 14β-propionyloxy. Accordingly, the structure of **1** was deduced as shown in Figure 2, and was named pierisoid C.

Compound **2** was obtained as colorless oil with a molecular formula of C_23_H_36_O_8_, as determined by a combination of HR-EI-MS and NMR spectra (including ^1^H, ^13^C, and DEPT) (Figures S7–S2 in supporting information). The resemblance of the NMR spectra of **2** (Table 1) with those of **1** disclosed that **2** was another grayanane diterpenoid structurally similar to **1**. The major difference was the replacement of a methylene carbon in **1** by an oxygen-occurring methine in **2** (δ_C_ 78.2), suggesting that either C-6, or C-11, or C-12 of **2** was oxygenated. In the HMBC spectrum of **2**, the HMBC correlations from 5-OH to the methine carbon at δ_C_ 78.2 indicated that this methine was ascribable to C-6 (Figure 3b). Carefully comparison of ^13^C-NMR spectral data of **2** with those of **1** (Table 1) obviously found that the upfield-shift of C-15 (δ_C_ 68.6) and C-16 (δ_C_ 61.3) in **2**, indicated an oxygen bridge, was formed between C-15 and C-16; this was supported by the HR-EI-MS spectrum. In the ROESY spectrum of **2**, the correlations of Me-17 with H-15; of 3-OH and 5-OH with Me-18; and of 5-OH with 6-OH and H-7 indicated that 3-OH, 5-OH, 6-OH, H-7, H-15, and Me-17 were in the same β-orientation (Figure 3e). Consequently, the structure of **2** was determined as shown in Figure 2 and was named pierisoid D.

Compound **3**, colorless crystals, has a molecular formula of C_31_H_42_O_14_, as determined by a combination of HR-EI-MS and NMR spectra (including ^1^H-, ^13^C-, and DEPT) (Figures S13–S18 in supplementary data). Its spectroscopic data were very similar to those of secorhodomollolide B, a 3,4-secograyanane diterpenoid also isolated from *P. formosa* [27]. The only difference between them was that the terminal double bond between C-4 (δ_C_ 146.0) and C-18 (δ_C_ 116.7) in secorhodomollolide B was replaced by a 4,18-oxirane group (δ_C_ 62.5 and 52.8) in **3**, which was confirmed by the HMBC correlations from Me-19 (δ_H_ 1.34, s) to C-4, C-5 (δ_C_ 87.5) and C-18 (Figure 3c). Such an oxirane moiety has also been found in pierisoid A, another 3,4-secograyanane diterpenoid we reported from the flowers of *P. formosa* [20]. In the ROESY spectrum of **3**, the correlations of Me-19 with H-1, and of Me-20 with H-7 indicated that Me-19 and H-1 were in α-orientation and Me-20 coupled with H-7 were in β-orientation (Figure 3f). Therefore, compound **3** was identified as shown in Figure 2 and was named pierisoid E.

Ten known diterpenoids (Figure 2), namely, pierisformotoxin C (**4**) [24], secorhodomollolides C (**5**), D (**6**), and F (**7**) [28], asebotoxins I (**8**), II (**12**), IV (**10**), and VIII (**11**) [26,29], pieristoxin I (**9**) [29], and pierisformosin (**13**) [30] were also isolated from *P. formosa* and were identified by comparison of their spectroscopic data with those reported in the literature.

### 2.2. Antifeedant Activity of Compounds ***1**, **3**, **4**,* and ***10***

The antifeedant activity of **1**, **3**, **4** and **10** against the generalist insect herbivore, beet armyworm (*Spodoptera exigua*), was assayed as previously described [31,32,33]. Compounds **1**, **3** and **10** were found to be potential deterrents of the beet armyworm, with EC_50_ values of 10.91, 33.89 and 6.58 μg/cm^2^, respectively. It seems that the antifeedant activity of grayanane diterpenoids may be reduced with the increase of degree of esterification. Although less active than the commercial neem oil containing 1% azadirachtin (EC_50_ = 3.71 μg/cm^2^) (Table 2), the significant antifeedant activity of these individual compounds and the overall effect they might have suggest a defensive role of grayanane diterpenoids for *P. formosa* against insect herbivores.

## 3. Discussion

Compounds **1**–**3** are highly oxygenated grayanane diterpenoids, which occur extensively in the plants of Ericaceae and exhibit remarkable biological activities, such as antifeedant and insecticidal activities. Compound **2** is a grayanane-type diterpenoid with an unprecedented 15,16-epoxy group in the grayanoids family. Compound **3**, a 3,4-seco-grayanane diterpenoid, possesses a 4,18-epoxy substituent, which is also unusual in nature. Compared to compounds **1** and **3**, **10** displayed more significant antifeedant activity against *S. exigua*, which was possibly attributed to the integrity of the A ring.

## 4. Materials and Methods

### 4.1. General Experimental Procedures

Melting points were recorded on an Aisey YLD-6000 instrument and are uncorrected. Column chromatography was performed on 200–300 mesh silica gel (Qingdao Marine Chemical Factory, Qingdao, China). Optical rotations were measured on a Horiba-SEAP-300 spectropolarimeter (Horiba, Tokyo, Japan). UV spectral data were obtained on a Shimadzu-210A double-beam spectrophotometer (Shimadzu, Tokyo, Japan). IR spectra were recorded on a Bruker-Tensor-27 spectrometer with KBr pellets (Bruker Optics, Ettlingen, Germany). NMR experiments were carried out on either a Bruker AV-400 or a DRX-500 spectrometer with tetramethyl silane (TMS) as an internal standard (Bruker, Karlsruhe, Germany). MS were recorded on a VG-Auto-Spec-3000 spectrometer (Waters Corp., Milford, MA, USA). TLC (Thin Layer Chromatography) spots were visualized under UV light, by dipping into 10% H_2_SO_4_ in EtOH followed by heating. All solvents including petroleum ether (60−90 °C) were distilled before use.

### 4.2. Plant Materials

The flowers of *P. formosa* were collected at Qu Jing, Yunnan province, China, in March 2008. The plant material was identified by Dr. Sheng-Hong Li.

### 4.3. Extraction and Isolation

Air-dried *P. formosa* flowers (3.5 kg) were powdered and extracted with MeOH (3 × 5 L) at room temperature. The extract was concentrated under reduced pressure and then partitioned between H_2_O and EtOAc (1:1). The organic layer (EtOAc part) was concentrated and the residue (594 g) was purified by silica gel column chromatography with solvent mixtures of CHCl_3_−Me_2_CO (1:0, 9:1, 8:2, 7:3, 1:1 and 0:1) to afford six fractions. Fraction 2 (45.0 g, CHCl_3_−Me_2_CO, 9:1) was repeatedly chromatographed on silica gel (CHCl_3_−Me_2_CO, 10:1; petroleum ether−Me_2_CO, 4:1), yielding subfractions A1−A3. Subfraction A1 (7.9 g) was further purified by silica gel column chromatography using petroleum ether-isopropanol (20:1), followed by purification on Sephadex LH-20 columns (CHCl_3_−MeOH, 1:1) to obtain compounds **4** (6 mg), **5** (5 mg), and **7** (81 mg). Subfraction A2 (6.65 g) was repeatedly separated by silica gel column chromatography eluting with petroleum ether-acetone (6:1) and finally was purified by Sephadex LH-20 columns (CHCl_3_−MeOH, 1:1; Me_2_CO) to afford compounds **1** (27 mg), **3** (3 mg), and **6** (6 mg). Subfraction A3 (10.2 g) was separated by silica gel column chromatography eluting with petroleum ether−ethyl acetate (5:1) and petroleum ether−isopropanol (12:1), respectively, and then similarly purified by Sephadex LH-20 columns (CHCl_3_−MeOH, 1:1; Me_2_CO) to give **8** (78 mg) and **9** (7 mg). Fraction 3 (4.2 g, CHCl_3_−Me_2_CO, 8:2) was separated on a MCI (Middle Chromatogram Isolated) gel column employing solvent mixtures of MeOH−water (6:4, 7:3, 8:2, 9:1, and 1:0), and the resulting subfractions 2 and 3 (7:3 and 8:2 MeOH−water, respectively) were repeatedly chromatographed on silica gel (CHCl_3_-EtOAc, 4:1; petroleum ether−isopropanol, 10:1) and Sephadex LH-20 (MeOH; Me_2_CO) columns to yield compounds **2** (18 mg), **10** (45 mg), **11** (8 mg), **12** (9 mg) and **13** (6 mg).

*Pierisoid C* (**1**): Colorless crystals; mp 220–222 °C; ${\left[\alpha \right]}_{D}^{24.8}$= +14.3 (*c* = 0.2, MeOH); UV (MeOH) λ_max_ (log *ε*): 209 (1.98), 193 (1.60) nm; IR (KBr) ν_max_: 3550, 3484, 2976, 2942, 1752, 1729, 1408, 1373, 1261, 1153, 1047 cm^−1^; ESI-MS: *m*/*z* 507 (50) [M + Na]^+^; HR-ESI-MS: *m*/*z* 507.2567 [M + Na]^+^ (calcd. for C_25_H_40_O_9_Na, 507.2570); ^1^H- and ^13^C-NMR data: see Table 1.

*Pierisoid D* (**2**): Colorless oils; ${\left[\alpha \right]}_{D}^{23.8}$= +16.7 (*c* = 0.1, MeOH); UV (MeOH) λ_max_ (log *ε*): 202 (2.48) nm; IR (KBr) ν_max_: 3441, 3432, 2971, 2939, 1735, 1730, 1630, 1382, 1179, 1049, 1025 cm^−1^; ESI-MS: *m*/*z* 463 (88) [M + Na]^+^; HR-EI-MS: *m*/*z* 440.2420 (calcd. for C_23_H_36_O_8_, 430.2410); ^1^H- and ^13^C-NMR data: see Table 1.

*Pierisoid E* (**3**): Colorless crystals; mp 271–272 °C; ${\left[\alpha \right]}_{D}^{23.6}$= +12.6 (*c* = 0.1, MeOH); UV (MeOH) λ_max_ (log *ε*): 202 (2.29) nm; IR (KBr) ν_max_: 3441, 2942, 1787, 1734, 1631, 1372, 1264, 1237, 1053 cm^-1^; ESI-MS: *m*/*z* 661 (68) [M + Na]^+^; HR-EI-MS: *m*/*z* 638.2560 (calcd. for C_31_H_42_O_14_, 638.2575); ^1^H- and ^13^C-NMR data: see Table 1.

### 4.4. Antifeedant Activity

Beet armyworms (*Spodoptera exigua*) were purchased from the Pilot-Scale Base of Bio-Pesticides, Institute of Zoology, Chinese Academy of Sciences. A modified dual-choice bioassay was performed for an antifeedant test as previously described [20,31,32,33]. The larvae were reared on an artificial diet under a controlled photoperiod (light:dark, 12:8 h) and temperature (25 ± 2 °C). The larvae were starved for 3−4 h before each bioassay. Fresh leaf disks were cut from *Brassica chinensis*, using a cork borer (1.1 cm in diameter). The treated leaf disks were painted with 10 μL of the test compound in acetone, and control leaf disks were treated with the same amount of acetone. After air drying, the tested and control leaf disks were set in alternating position in the same Petri dish (90 mm in diameter), with moistened filter paper at the bottom. Two-thirds of the instars were placed at the center of the Petri dish. Five replicates were run for each treatment. After feeding for 24 h, the areas of leaf disks consumed were measured. The antifeedant index (AFI) was calculated according to the following formula: AFI = [(*C* − *T*)/(*C* + *T*)] × 100, where *C* and *T* represent the control and treated leaf areas consumed by the insect. The insect antifeedant potency of the test compound was evaluated in terms of the EC_50_ value, which was determined by probit analysis for each insect species.

## 5. Conclusions

Terpenoids play an important role in natural product chemistry and biology, such as antifungal and insecticidal activities [34]. Secondary metabolites, such as the grayanane diterpenoids that occur extensively in the plants of Ericaceae, are fascinating for their remarkable toxicity, as well as their significant antifeedant and insecticidal activity. In the current study, three new grayanane diterpenoids, pierisoids C–E (**1**‒**3**), as well as 10 known ones (**4**‒**13**), were identified from the flowers of *P. formosa* via their extensive 1D and 2D NMR spectroscopic data analyses. Notably, compounds **1**, **3** and **10**, especially **10**, exhibited obvious antifeedant activity against the beet armyworm (*S. exigua*), suggesting that these diterpenoids were important defensive substances in *P. formosa* against natural enemies.

## Figures and Tables

**Table 1:** ^1^H- (400 MHz) and ^13^C- (100 MHz) NMR spectroscopic data of compounds **1**–**3** in acetone-*d*_6_ (δ (ppm), *J* (Hz)).

No.	1 ^a^		2 ^b^		3	
	δ_H_	δ_C_	δ_H_	δ_C_	δ_H_	δ_C_
1	2.74 dd (11.6, 5.2)	50.9 d	2.77 m	50.9 d	3.08 m	52.6 d
2a 2b	2.06 m 2.27 m	35.4 t	2.05 m 2.22 m	35.9 t	2.47 m 2.73 dd (18.9, 11.8)	32.8 t
3	3.60 t (5.0)	82.5 d	3.53 dd (1.5, 5.1)	83.0 d		174.0 s
4		51.4 s		52.3 s		62.5 s
5		84.1 s		83.1 s		87.5 s
6	2.05 m (2H)	34.0 t	3.91 overlap	78.2 d	6.04 d (9.6)	69.8 d
7	3.62 t (5.0)	72.4 d	3.41 overlap	78.3 d	5.23 d (9.7)	68.7 d
8		54.5 s		53.8 s		56.1 s
9	2.08 m	54.6 d	2.08 m	49.9 d	2.32 m	47.0 d
10		78.4 s		77.7 s		77.4 s
11a 11b	1.58 m 1.90 m	21.8 t	1.71 m 1.81 m	21.6 t	1.98 m 2.03 m	21.1 t
12a 12b	1.58 m 2.23 m	27.0 t	1.68 m 2.35 m	28.1 t	1.74 m 2.08 m	25.8 t
13	2.05 overlap	52.9 d	2.24 m	47.6 d	3.10 overlap	46.4 d
14	5.59 s	82.1 d	5.52 s	76.4 d	6.43 s	79.3 d
15	4.93 s	85.0 d	3.26 s	68.6 d	5.27 s	86.2 d
16		79.6 s		61.3 s		88.4 s
17	1.27 s (3H)	22.8 q	1.45 s (3H)	14.6 q	1.55 s (3H)	19.4 q
18	1.11 s (3H)	18.5 q	1.23 s (3H)	19.3 q	2.49 d (5.0) 2.98 d (4.9)	52.8 t
19	0.91 s (3H)	23.0 q	0.97 s (3H)	23.2 q	1.34 s (3H)	17.4 q
20	1.41 s (3H)	27.8 q	1.41 s (3H)	28.2 q	1.51 s (3H)	33.6 q
6-OAc					2.02 s (3H)	20.6 q
						169.4 s
7-OAc					2.10 s (3H)	21.6 q
						169.8 s
14-OPr	1.11 t (3H, 7.6) 2.85 m (2H)	9.6 q	1.08 t (3H, 7.6) 2.27 m (2H)	9.5 q	1.08 t (3H, 7.5) 2.36 m (2H)	9.1 q
		28.4 t		28.5 t		28.2 t
		173.4 s		173.8 s		174.0 s
15-OAc	2.10 s (3H)	21.0 q			1.98 s (3H)	20.8 q
		170.3 s				171.6 s
16-OAc					1.98 s (3H)	22.7 q
						169.8 s

**^a^** Hydroxyl groups of **1**: δ_H_ 4.20 (d, *J* = 5.4 Hz, 3-OH), 3.39 (s, 10-OH), 3.06 (s, 16-OH); **^b^** Hydroxyl groups of **2**: δ_H_ 3.91 (overlap, 3-OH), 3.64 (s, 5-OH), 3.51 (s, 10-OH), 3.41 (overlap, 6-OH), 3.34 (d, *J* = 7.8 Hz, 7-OH).

**Table 2:** Antifeedant activity of compounds **1**, **3**, **4**, and **10** against *Spodoptera exigua.*

Compound	Molecular Formula	*m*/*z*	EC_50_ (µg/cm^2^)
**1**	C_25_H_40_O_9_	484	10.91
**3**	C_31_H_42_O_14_	638	33.89
**4**	C_33_H_46_O_15_	682	NA
**10**	C_23_H_38_O_8_	442	6.58
Neem oil	-	-	3.71

NA = Not active; “-” = No (Because neem oil is a mixture).

## Supplementary Materials

Supplementary Materials are available online. 1D (^1^H- and ^13^C-) and 2D NMR spectra of compounds **1**–**3** are available online.
