# Supplementary material for: New Antifeedant Grayanane Diterpenoids from the Flowers of Pieris formosa
Source: Molecules. 2017 Aug 31;22(9):1431. doi: 10.3390/molecules22091431 (PMC6151510; doi:10.3390/molecules22091431)
Supplement: Supplementary file 1 [file molecules-22-01431-s001.pdf]

Supplementary Data

New antifeedant grayanane diterpenoids from the flowers of *Pieris Formosa*

Chun-Huan Li[1], Shi-Hong Luo[2], Sheng-Hong Li, [2,*] and Jin-Ming Gao[1,*]

[1] Shanxi Key Labotory of Natural Products &Chemical Biology, College of Chemistry & Pharmacy, Northwest A&F University, Yangling 712100, People's Republic of China

[2] State Key Laboratory of Phytochemistry and Plant Resources in West China, Kunming Institute of Botany, Chinese Academy of Sciences, Lanhei Road 132, Kunming 650201, People's Republic of China

* Correspondence: jinminggao@nwsuaf.edu.cn(J.M.G.); Tel.: +86-29-87092515; shli@mail.kib.ac.cn (S.-H. Li).Tel/Fax: +86 871 65223035.

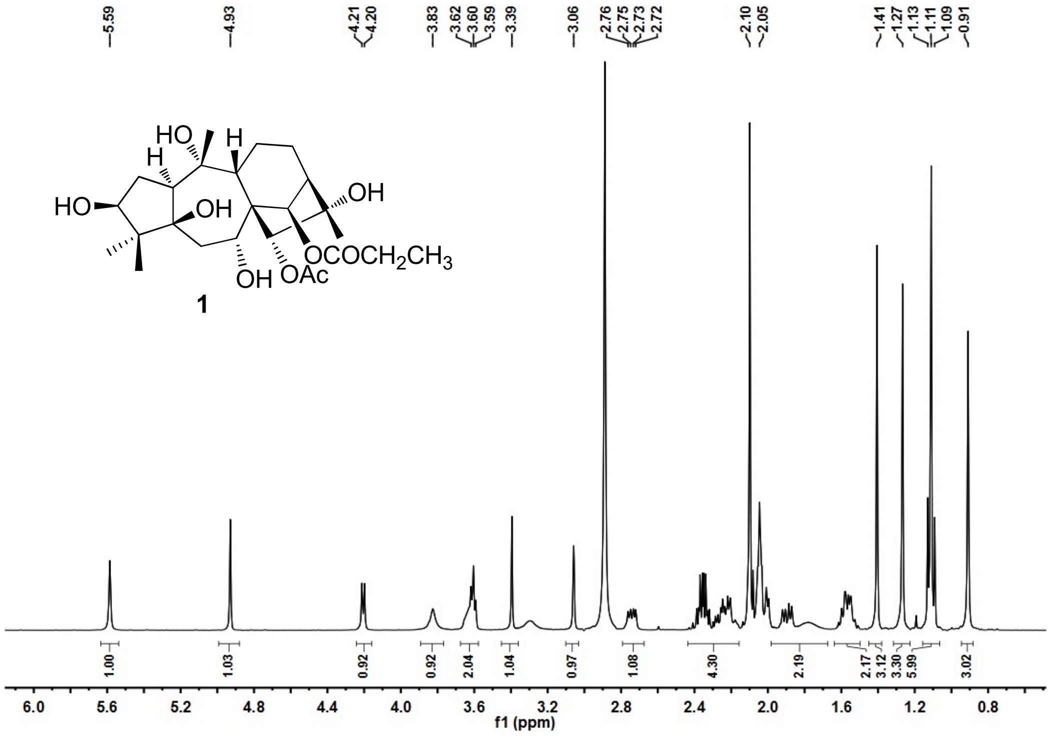

Figure S1. ^{1}H spectrum of PierisoidC (**1**) in acetone-d_6

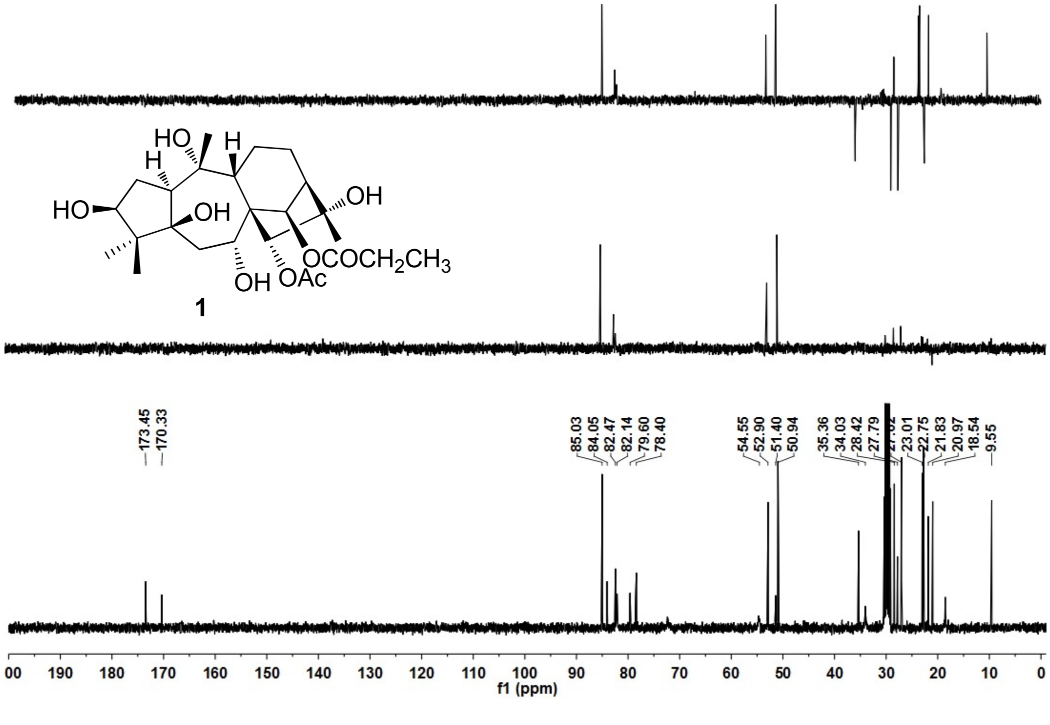

Figure S2. ^{13}C NMR spectrum of Pierisoid C (**1**) in acetone-d_6

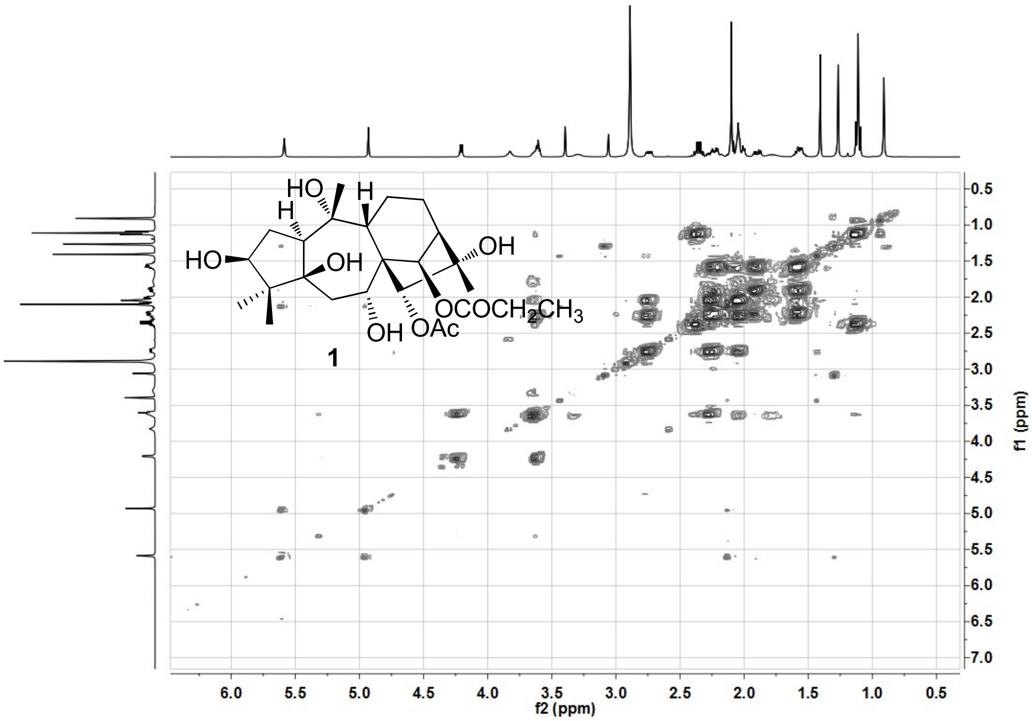

Figure S3. ^1H-^1H COSY spectrum of Pierisoid C (**1**) in acetone-d_6

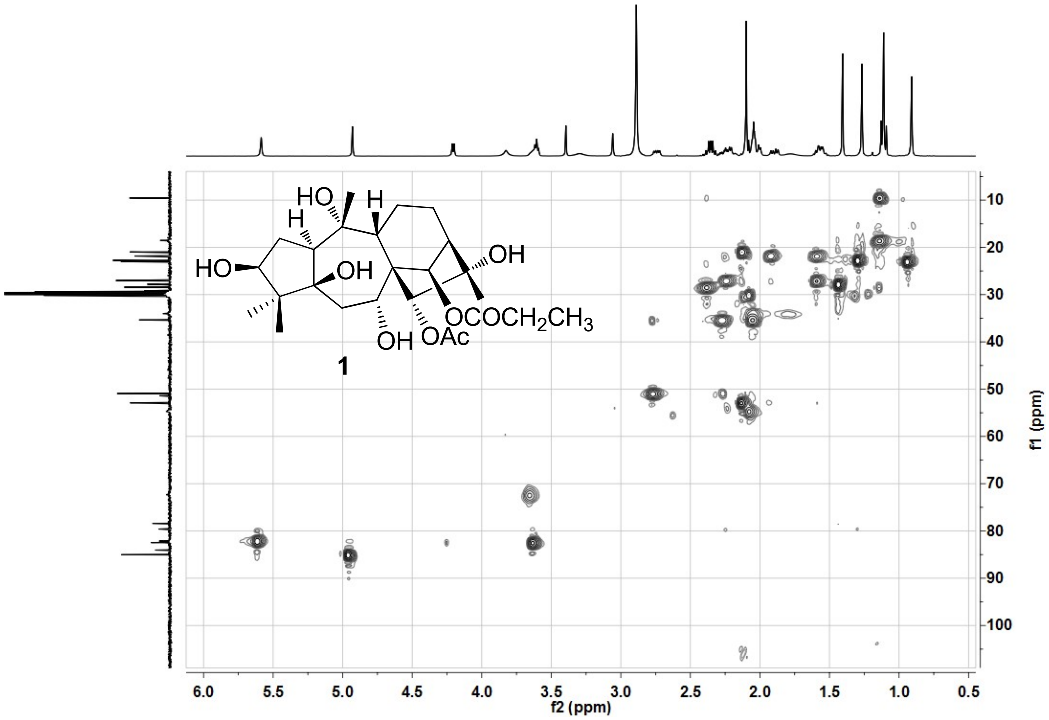

Figure S4. HSQC spectrum of Pierisoid C (**1**) in acetone-d_6

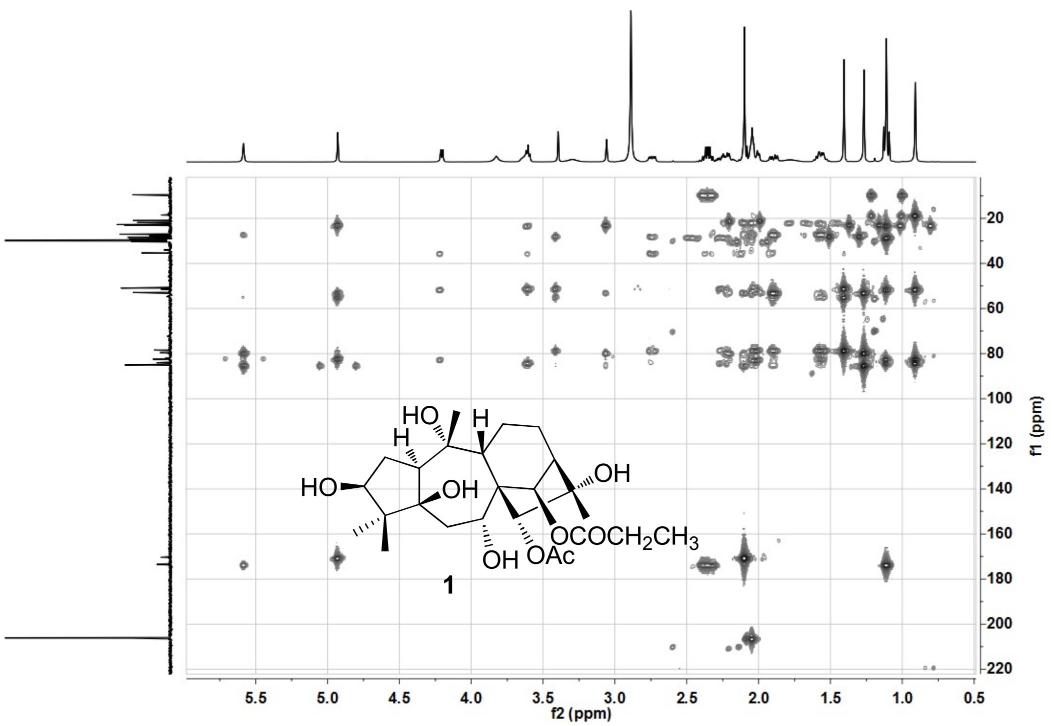

Figure S5. HMBC spectrum of Pierisoid C (**1**) in acetone-d_6

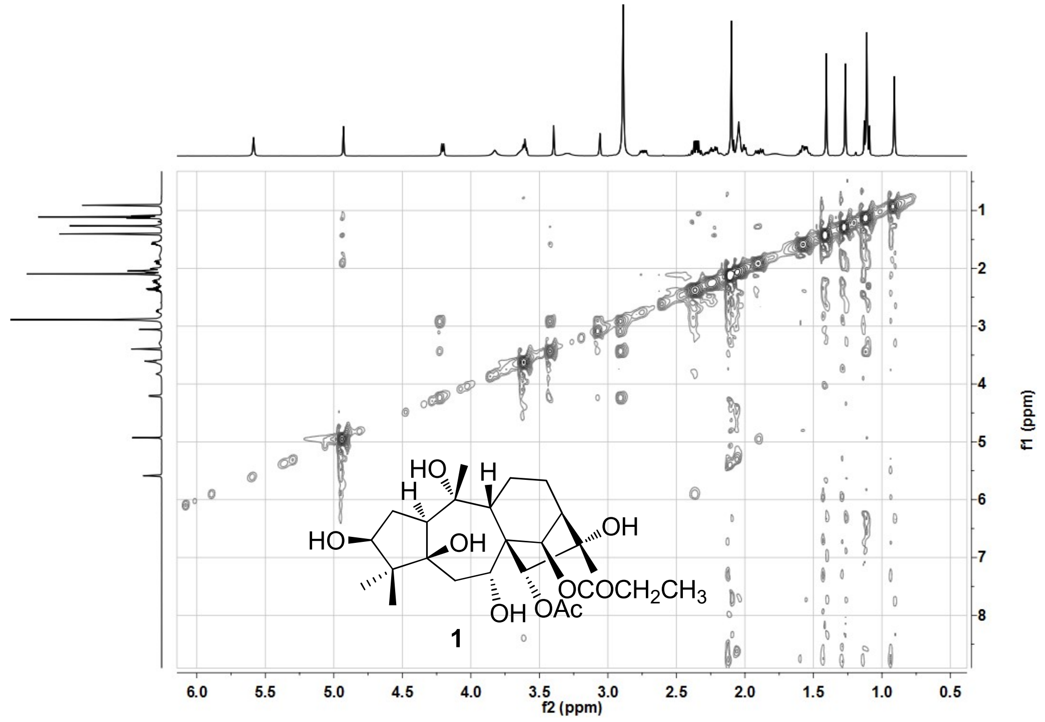

Figure S6. ROESY spectrum of Pierisoid C (**1**) in acetone-d_6

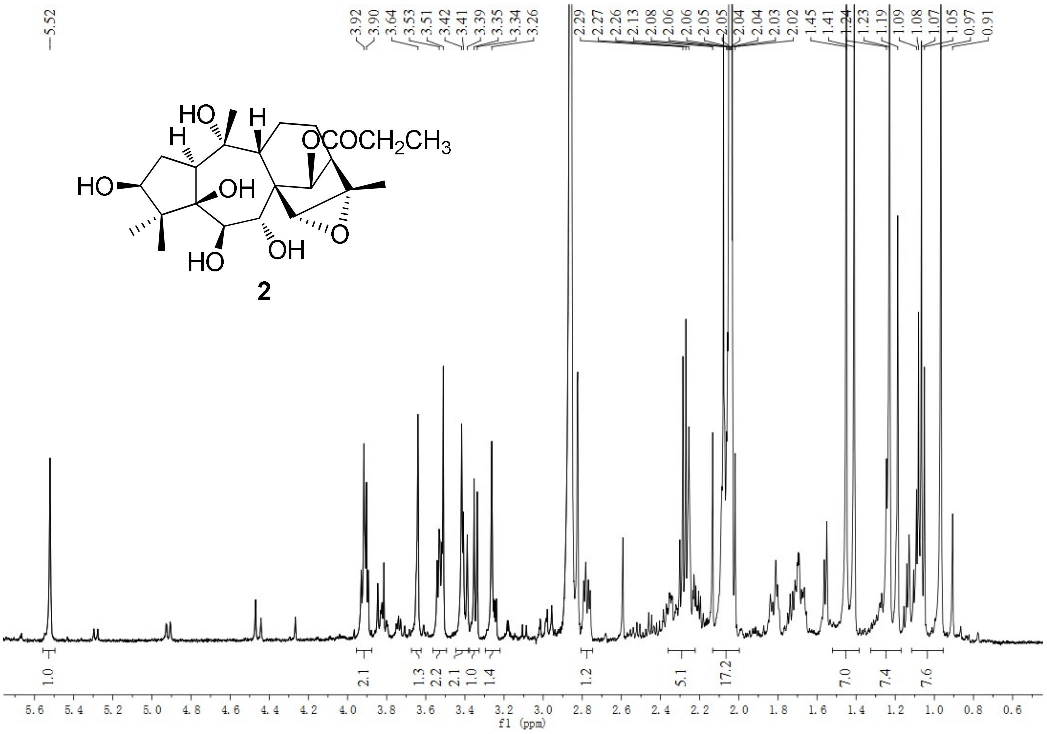

Figure S7. ^{1}H spectrum of Pierisoid D (**2**) in acetone-d_6

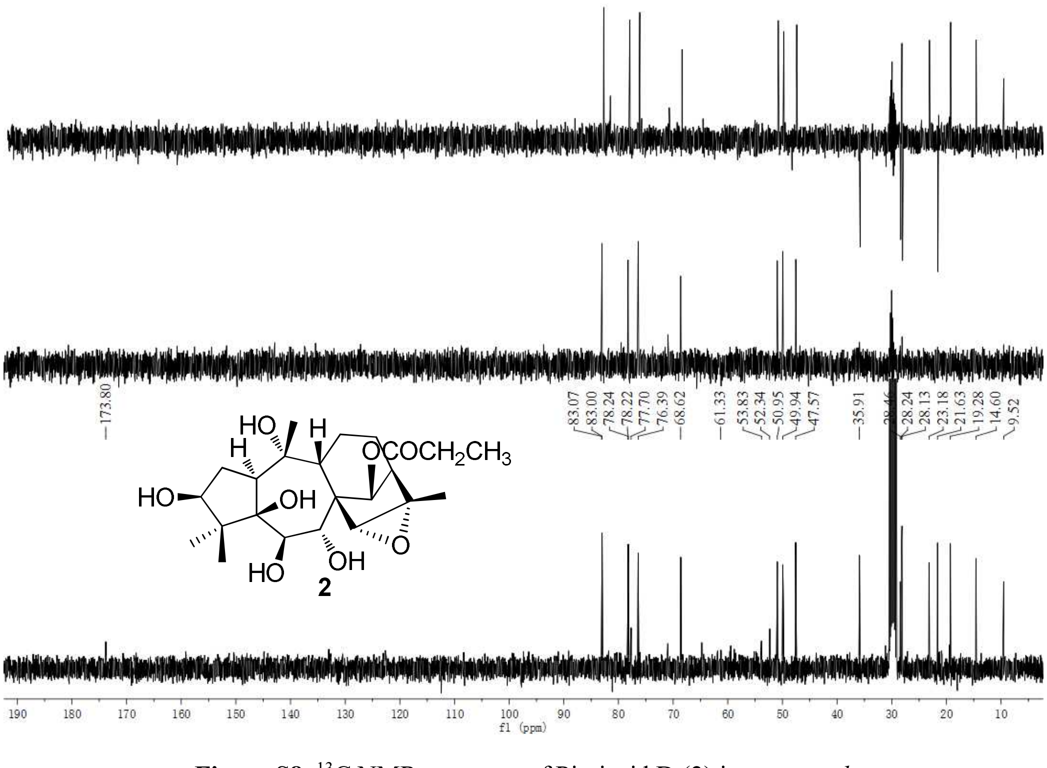

Figure S8. ^{13}C NMR spectrum of Pierisoid D (**2**) in acetone-d_6

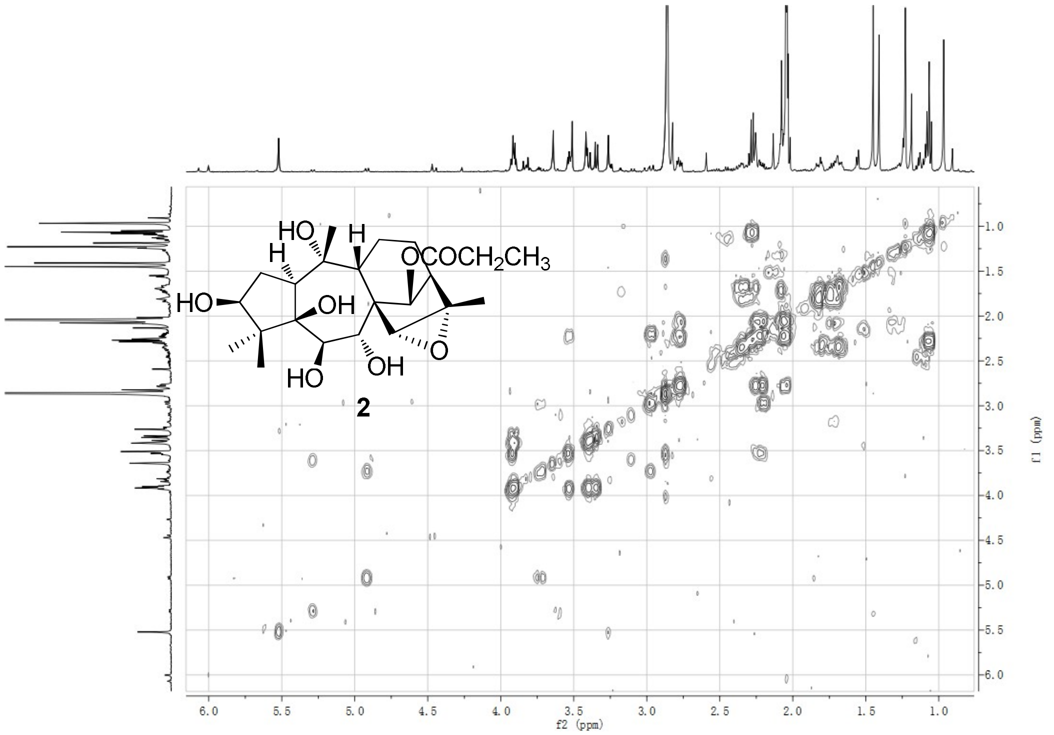

Figure S9. ^{1}H-^{1}H COSY spectrum of Pierisoid D (**2**) in acetone-d_6

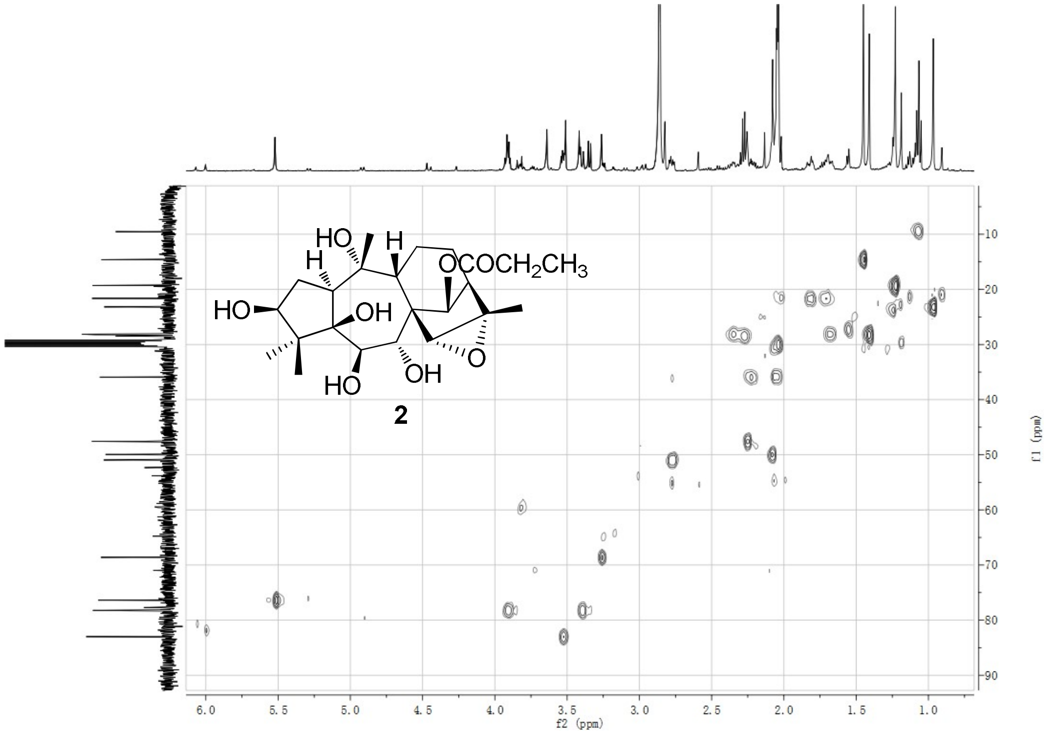

Figure S10. HSQC spectrum of Pierisoid D (**2**) in acetone-d_6

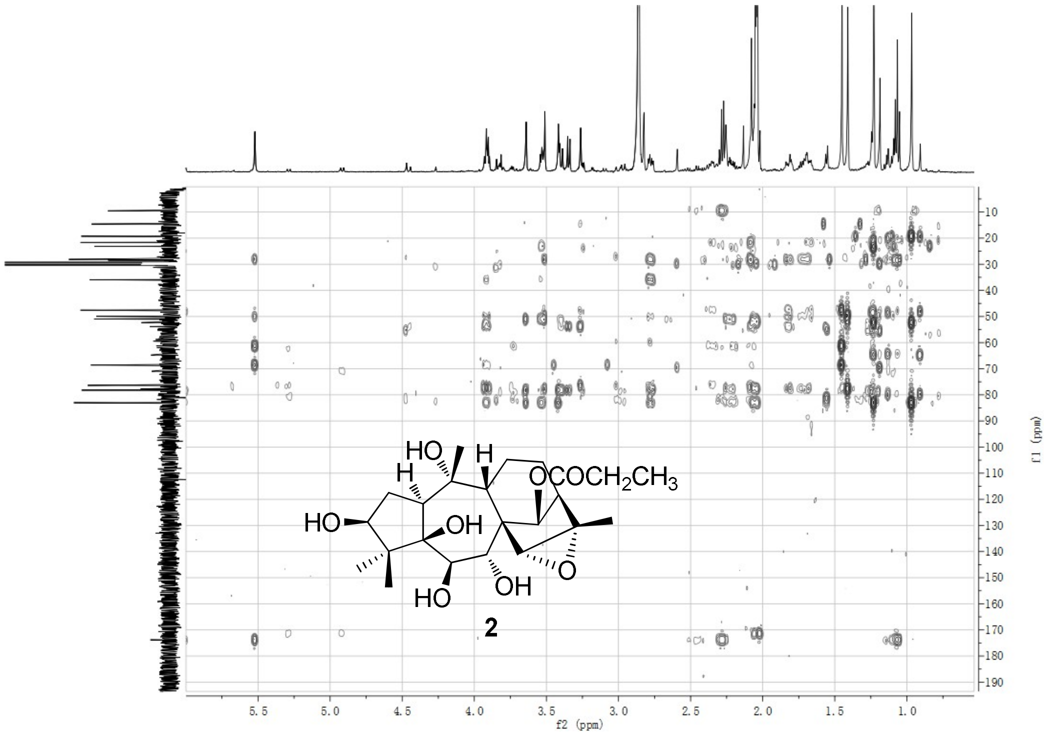

Figure S11. HMBC spectrum of Pierisoid D (**2**) in acetone-*d*$_6$

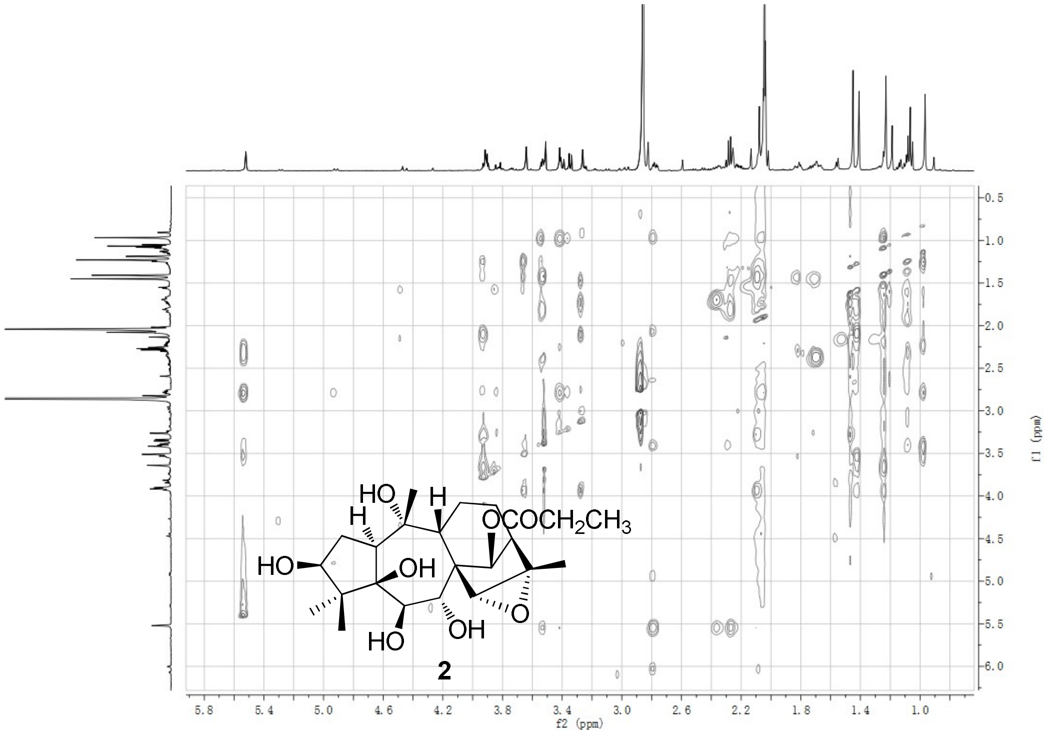

Figure S12. ROESY spectrum of Pierisoid D (**2**) in acetone-*d*$_6$

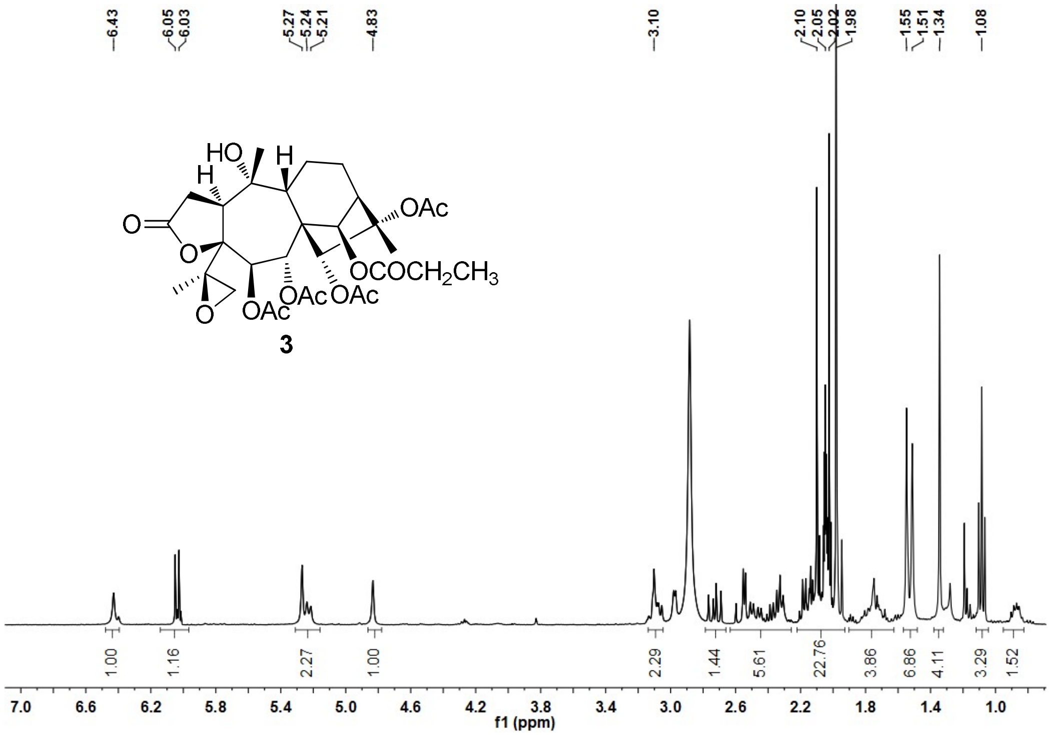

Figure S13. ^{1}H spectrum of Pierisoid E (**3**) in acetone-d_6

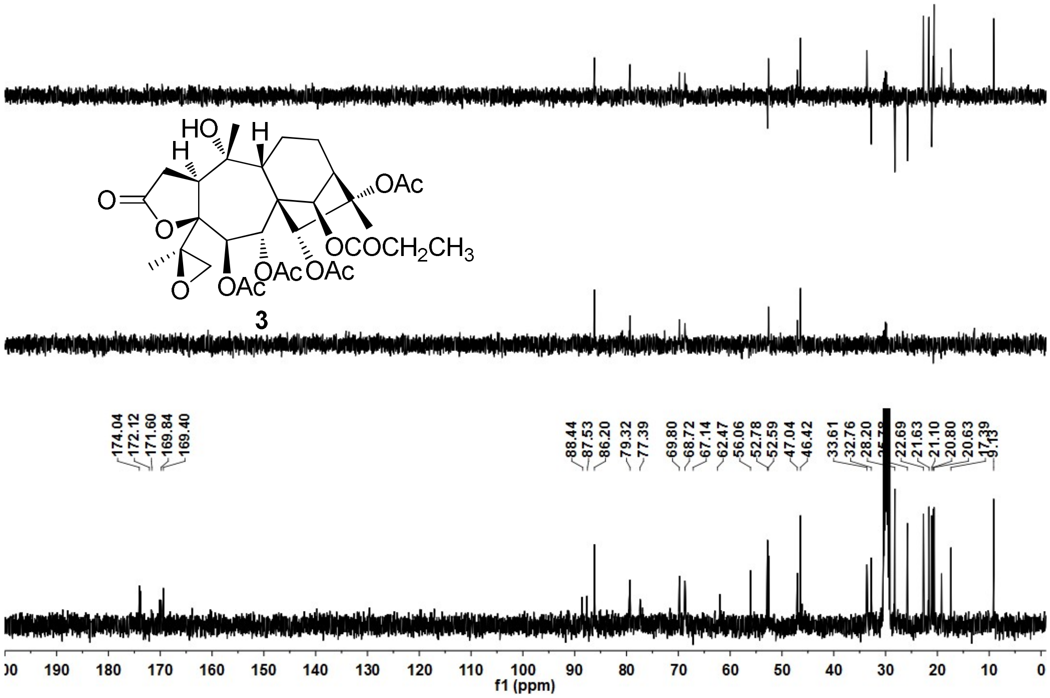

Figure S14. ^{13}C NMR spectrum of Pierisoid E (**3**) in acetone-d_6

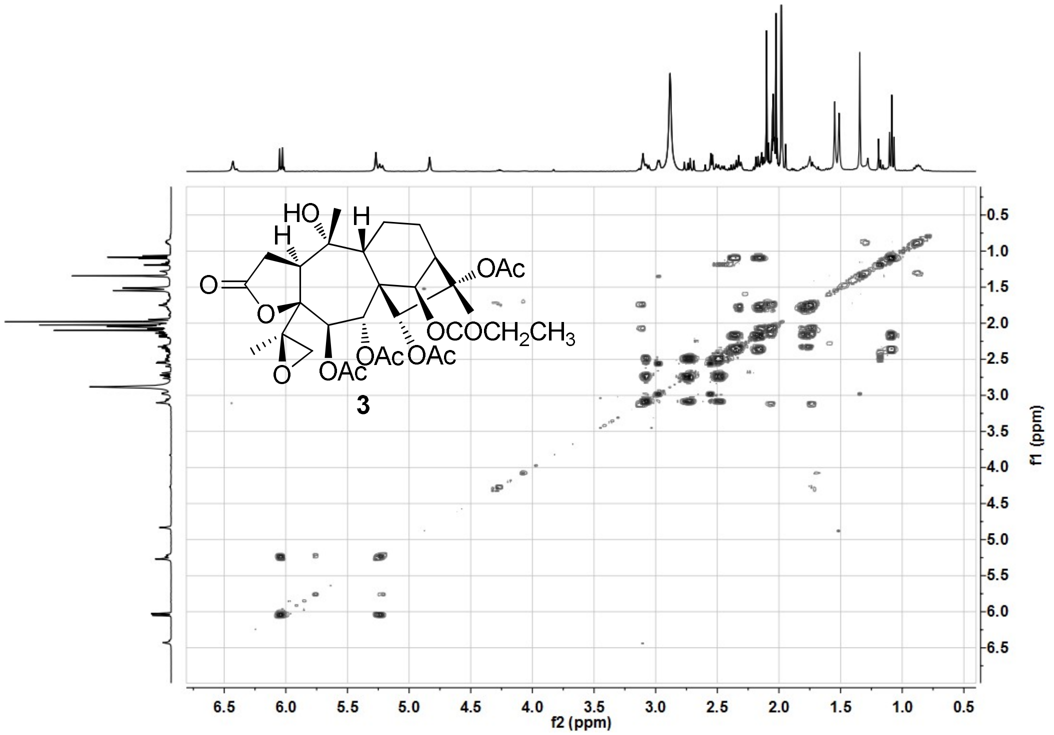

Figure S15. ^{1}H-^{1}H COSY spectrum of Pierisoid E (**3**) in acetone-d_6

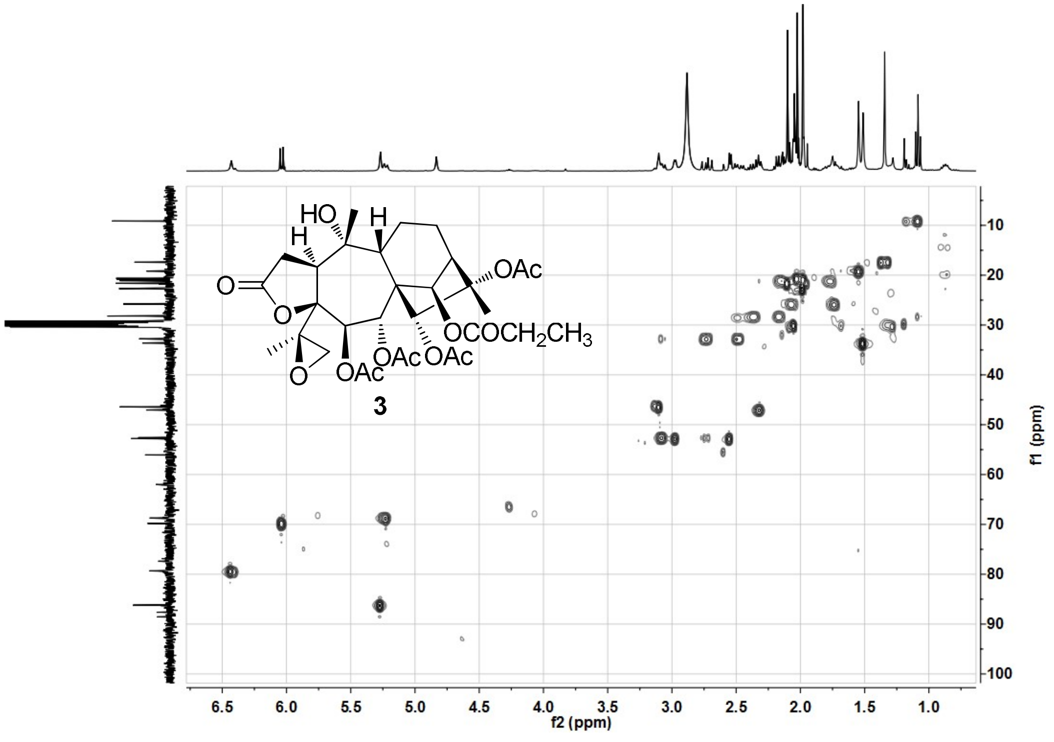

Figure S16. HSQC spectrum of Pierisoid E (**3**) in acetone-d_6

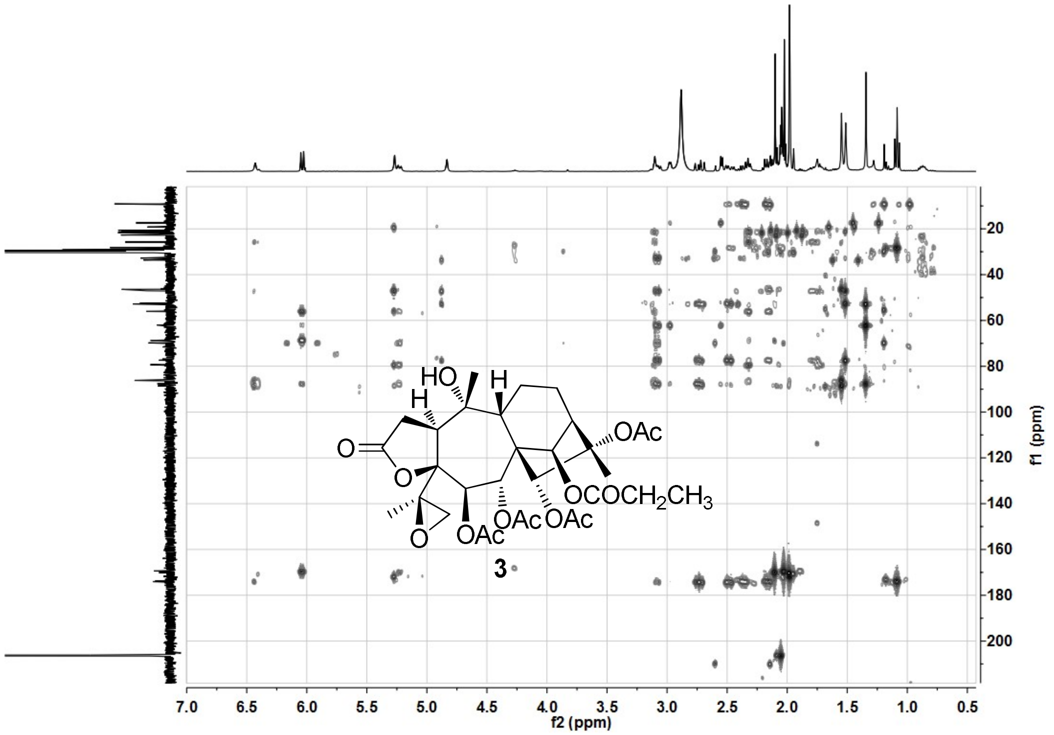

Figure S17. HMBC spectrum of Pierisoid E (**3**) in acetone-d_6

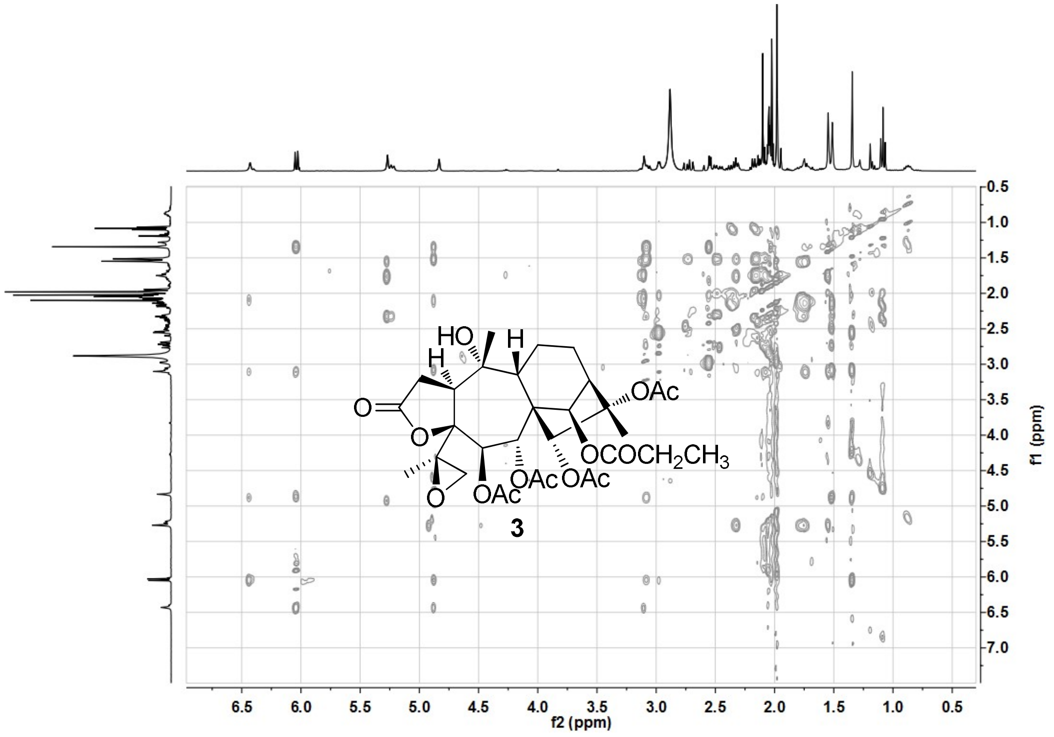

Figure S18. ROESY spectrum of Pierisoid E (**3**) in acetone-d_6
